# Safety and tolerability of liquid amikacin in antibiotic-loaded bone cement – a case series

**DOI:** 10.5194/jbji-6-147-2021

**Published:** 2021-04-26

**Authors:** Don Bambino Geno Tai, Nathan J. Brinkman, Omar Abu Saleh, Douglas R. Osmon, Matthew P. Abdel, Christina G. Rivera

**Affiliations:** 1 Division of Infectious Diseases, Department of Medicine, Mayo Clinic, Rochester, MN 55905, USA; 2 Department of Pharmacy, Mayo Clinic, Rochester, MN 55905, USA; 3 Department of Orthopedic Surgery, Mayo Clinic, Rochester, MN 55905, USA

## Abstract

High-dose liquid antibiotics are uncommon in bone cement. We present a case series of patients in which up to 16 mL of liquid amikacin (250 mg mL${}^{-1}$) was successfully incorporated into bone cement to treat periprosthetic joint infections. We did not observe adverse drug reactions definitively attributed to its use.

## 1 Introduction

Antibiotic-loaded bone cement (ALBC) is ubiquitous in orthopedic surgery. Bone cement anchors a prosthesis during primary and revision total knee arthroplasties, total hip arthroplasties (THA), and various upper-extremity arthroplasties. ALBC is commonly used as an adjunct in the treatment of periprosthetic joint infections (PJIs) and other infections of the musculoskeletal (MSK) system (Schwarz et al., 2021). In cases of two-stage exchange arthroplasty for PJI, ALBC is used as a spacer for stability while at the same time acting as a local drug delivery system (Athans et al., 2017). Aminoglycosides such as tobramycin and gentamicin are more commonly used in ALBC. Amikacin is also an aminoglycoside with a narrow therapeutic index and large pharmacokinetic variability but rarely incorporated into ALBC. It is a staple of non-tuberculous mycobacterial (NTM) MSK infection treatment regimens whenever the susceptibility profile is favorable (Goldstein et al., 2019). NTM MSK infections are challenging to treat and typically require months of combination intravenous (IV) and oral antibiotics with the potential for significant adverse reactions, particularly nephrotoxicity and ototoxicity from IV aminoglycosides.

Amikacin in bone cement is an attractive option as an adjuvant treatment for NTM MSK infections. The powdered formulation of antimicrobials is preferred due to evidence of reducing biochemical strength when using liquid formulations (Athans et al., 2017). However, a powdered formulation of amikacin is not commercially available and therefore not readily available to hospital pharmacies in the United States. We hypothesized that liquid amikacin is a suitable alternative and safe for integration into weight-bearing and non-weight-bearing ALBC. We aimed to describe our experience in incorporating liquid amikacin into ALBC for clinical use.

## 2 Methods

We performed a case series study including patients in whom liquid amikacin was used in antibiotic-loaded bone cement (ALBC) for PJI treatment. Bone cement was polymethyl methacrylate (PMMA). We used the institutional electronic medical record (EMR) reporting tool and pharmacy database to obtain an initial list of patients in which liquid amikacin, 250 mg mL${}^{-1}$, was dispensed for intraoperative use from 1 January 2013 to 31 December 2018. We investigated each case using the EMR record for clinical characteristics, indication, culture results, the operation performed, ALBC production, and adverse drug reactions (ADRs). Patients were followed up to the last visit in the EMR for a minimum of 2 years. Patients in whom the liquid amikacin was not used in ALBC were excluded. We used the World Health Organization–Uppsala Monitoring Center (WHO-UMC) definition for ADR and causality assessment system.

**Table 1:** Summary of patients in whom amikacin-loaded bone cement was used.

Pt.	Age/sex	Indication	Organism	Use	Antibiotics perbatch	Amikacin concentration, mL g${}^{-1}$ of PMMA (mL perbatch)${}^{1}$	IV and oral antibiotics	Adverse drug reaction	WHO-UMC causality criteria	Outcome
1	64/M	Right kneePJI	*Mycobacterium abscessus*	Temporary static spacerafter resectionarthroplasty	4 g amikacin 3 g vancomycin 3 g gentamicin	0.4 (16)	Cefoxitin, linezolid, tigecycline	AKI in1 year	Unlikely	Cure, successful reimplantation
2	64/F	Right hipPJI	*M. fortuitum*, *Actinomyces neuii*, *Peptoniphilus* species	Temporary static spacerafter resectionarthroplasty	4 g amikacin 0.2 g amphotericin B 4 g vancomycin	0.4 (16)	Imipenem, linezolid, moxifloxacin	None	N/A	Cure, successful reimplantation
3	77/F	Right kneePJI withfemoral osteomyelitis	*M. abscessus, Staphylococcus epidermidis,Corynebacterium* species	Local drugdelivery systemafter AKA	4 g amikacin 8 g cefoxitin	0.4 (16)	Amikacin, cefoxitin, clofazimine, tigecycline	AKI in3 months	Possible${}^{2}$	Cure
4	49/F	Left kneePJI	Culture-negative	Temporaryarticulatingspacer afterresection arthroplasty	1 g amikacin 0.15 g amphotericin B 5 g vancomycin	0.1 (4)	Cefazolin	None	N/A	Cure, successful reimplantation

${}^{1}$ One batch of PMMA equals 40 g; liquid amikacin concentration was 250 mg mL${}^{-1}$. ${}^{2}$ with concurrent use of IV amikacin.Abbreviations: Pt., patient; AKA, above-the-knee amputation; PMMA, polymethyl methacrylate; WHO-UMC, World Health Organization–Uppsala Monitoring Center; AKI, acute kidney injury; N/A, not applicable.

## 3 Case series presentation

There were four patients with PJI (three knees and one hip) in whom liquid amikacin was incorporated into ALBC. ALBC was used as a temporary spacer after resection arthroplasty in three patients, while in one patient it was solely used as a local drug delivery system. The ALBC was prepared by surgeons performing the procedure. The liquid monomer was typically added to the powder component of PMMA before the addition of antimicrobials. The median amount of liquid amikacin per one batch of PMMA (40 g) was 16 mL amikacin (4 g). Clinical characteristics, antibiotics used, and compounding information are available in Table 1.

Patient 1 was a 64-year-old male with stage III chronic kidney disease (CKD) referred to our institution for *Mycobacterium abscessus* right knee PJI after management at an outside institution failed. He underwent resection arthroplasty, and a static ALBC spacer was inserted. His activity was toe touch weight bearing following this surgery. He had 1 year of combination systemic antibiotic therapy, but his inflammatory markers remained elevated. Due to the concern for persistent infection, the spacer was resected, and systemic antibiotics were extended for 3 more months. He underwent delayed reimplantation 4 months later and was doing well at 2 years of follow-up.

Patient 2 was a 64-year-old female who underwent primary THA at an outside institution complicated by polymicrobial infection. She underwent resection arthroplasty with implantation of a static ALBC spacer at our institution. Four out of five periprosthetic tissue cultures from this surgery grew *Mycobacterium fortuitum*, *Actinomyces neuii*, and *Peptoniphilus* species. The spacer was exchanged with another ALBC spacer with amikacin. She was toe touch weight bearing with a walker after surgery. She completed 10 months of a combination antimycobacterial regimen and eventually had delayed reimplantation 19 months later. She remained infection free after 2 years of follow-up.

Patient 3 was a 77-year-old female who had multiple surgeries and courses of antibiotics for a right knee PJI with *Staphylococcus epidermidis,* *Corynebacterium* species, and *M. abscessus*. She presented to our institution for a second opinion. Due to inadequate soft tissue coverage and bone stock, she underwent above-the-knee amputation. There was residual osteomyelitis above the level of amputation and eight out of eight femur and periprosthetic tissue cultures grew *M. abscessus* and *Staphylococcus epidermidis*. Three months into systemic combination antibiotic therapy, she had persistent drainage. MRI showed a fluid collection adjacent to the amputation margin and persistent osteomyelitis of the right femoral intramedullary canal. She underwent further debridement with deposition of amikacin and cefoxitin-loaded PMMA beads in the intramedullary canal. Incorporation of liquid amikacin (6 mL) into one batch of calcium sulfate beads (10 mL) was also attempted, but it did not cure after 45 min. The mixture was applied as paste instead. The PMMA beads were removed after 3 months. She eventually completed a total of 9 months of antibiotic therapy and was doing well after 2 years of follow-up.

Patient 4 was a 49-year-old female referred to our institution for culture-negative left knee PJI. She underwent debridement, antibiotic therapy, and implant retention in an outside institution but continued to have left knee pain. She then had resection arthroplasty with an articulating ALBC spacer. The surgeon chose amikacin over standard gentamicin in ALBC. The patient had a documented allergy to gentamicin in the medical record, but it was unclear from the medical record review if this was the rationale for using amikacin in the ALBC. Her activity after surgery was partial weight bearing of around 18 kg (40 pounds) on the left lower extremity, progressing up to full weight bearing at 4 weeks. She had successful reimplantation after 2 months and remained infection free after 7 years.

### 3.1 Adverse drug reactions

Patient 1 developed acute kidney injury (AKI) 1 year after insertion of ALBC and 1 month after starting IV amikacin. The peak serum amikacin level was 38.6 $µg\;{\mathrm{mL}}^{-1}$ (normal 20–35 $µg\;{\mathrm{mL}}^{-1}$). The patient did not recover his previous kidney function but had stable CKD. Due to the timing and use of IV amikacin, we designated this ADR as WHO-UMC causality category of “Unlikely”.

Patient 3 developed AKI 3 months after insertion of ALBC. She was also started on IV amikacin at the same time and had a detectable serum amikacin trough level of 1.7 $µg\;{\mathrm{mL}}^{-1}$ (normal < 0.8 $µg\;{\mathrm{mL}}^{-1}$). We designated this ADR as WHO-UMC causality criteria of “Possible” due to reasonable time relationship to ALBC implantation but could also be explained by concurrent use of IV amikacin. Her AKI resolved upon discontinuation of IV amikacin.

The other patients did not develop AKI. There were no reports of ototoxicity symptoms such as hearing loss, vertigo, tinnitus, or progression of baseline symptoms after placement of ALBC. In three patients, who were on varying degrees of weight bearing, there were no fractures reported. There were no signs of local adverse effects such as rash, wound dehiscence, or delayed wound healing in all cases.

## 4 Conclusions

This study demonstrated that liquid amikacin could be incorporated into ALBC and used to treat MSK infections, particularly NTM PJI. Liquid amikacin was successfully incorporated into PMMA bone cement. We did not find any systemic or local ADRs definitively related to their use in our small number of patients. Nevertheless, we recommend that clinicians monitor kidney function and for signs and symptoms of ototoxicity. Serum amikacin levels can be measured if there are adverse events that occur, which could be related to amikacin. Liquid antibiotics were postulated to enhance antibiotics' elution into surrounding bone and tissue but negatively impacted mechanical stability (Anagnostakos and Meyer, 2017; Chang et al., 2014). We did not observe mechanical instability, although only one patient was instructed to be on full weight bearing, and two were on toe touch weight bearing. Since this is the first report of a small number of patients on the clinical use of liquid amikacin in bone cement, there are no safety comparisons available to us to draw from.

All four patients in the cohort had favorable outcomes. However, due to the lack of a control group, we cannot conclude this therapeutic intervention's effect on the cure of infection. A previous study showed that liquid amikacin eluted concentrations higher than the minimum inhibitory concentration (MIC) for 30 d, suggesting clinical utility (Ethell et al., 2000). More research is needed to address the efficacy of liquid amikacin in ALBC to treat various MSK infections in which amikacin is the preferred drug. ALBC with high-dose liquid amikacin can be used in clinical scenarios when structural integrity is not a concern, such as intramedullary osteomyelitis or septic arthritis in non-weight-bearing joints. Amikacin seems safe to use as PMMA spacers in weight-bearing joints in our small case series, but further safety information in a larger cohort of patients is warranted to estimate its definitive safety profile.

In conclusion, up to 16 mL of liquid amikacin (250 mg mL${}^{-1}$) was incorporated into ALBC to treat MSK infection in a small number of patients with a favorable outcome. More research is needed on the standardization of formulation and clinical efficacy and safety profile in a larger group of patients to make definitive conclusions regarding liquid amikacin's efficacy and safety in ALBC.

## Data Availability

Identifiable personal data cannot be accessed by the public under the Health Insurance Portability and Accountability Act of the United States.
